# Supplementary material for: The Effect of Magnetic Composites (γ-Al2O3/TiO2/γ-Fe2O3) as Ozone Catalysts in Wastewater Treatment
Source: Materials (Basel). 2022 Nov 28;15(23):8459. doi: 10.3390/ma15238459 (PMC9738670; doi:10.3390/ma15238459)
Supplement: Supplementary file 1 [file materials-15-08459-s001.zip › materials-2041176-supplementary.pdf]

The effect of magnetic composites (γ-Al_2O_3/TiO_2/γ-Fe_2O_3) as ozone catalysts in wastewater treatment

Cheng Wang [a], Guangzhen Zhou [a], Yanhua Xu [a, *] and Peng Yu [a]

[a] School of Environmental Science and Engineering, Nanjing Tech University, Nanjing, 211816, China

*Corresponding Author: Yanhua Xu

Fax: +86-025-58139652

E-mail: yanhuaxu18@hotmail.com

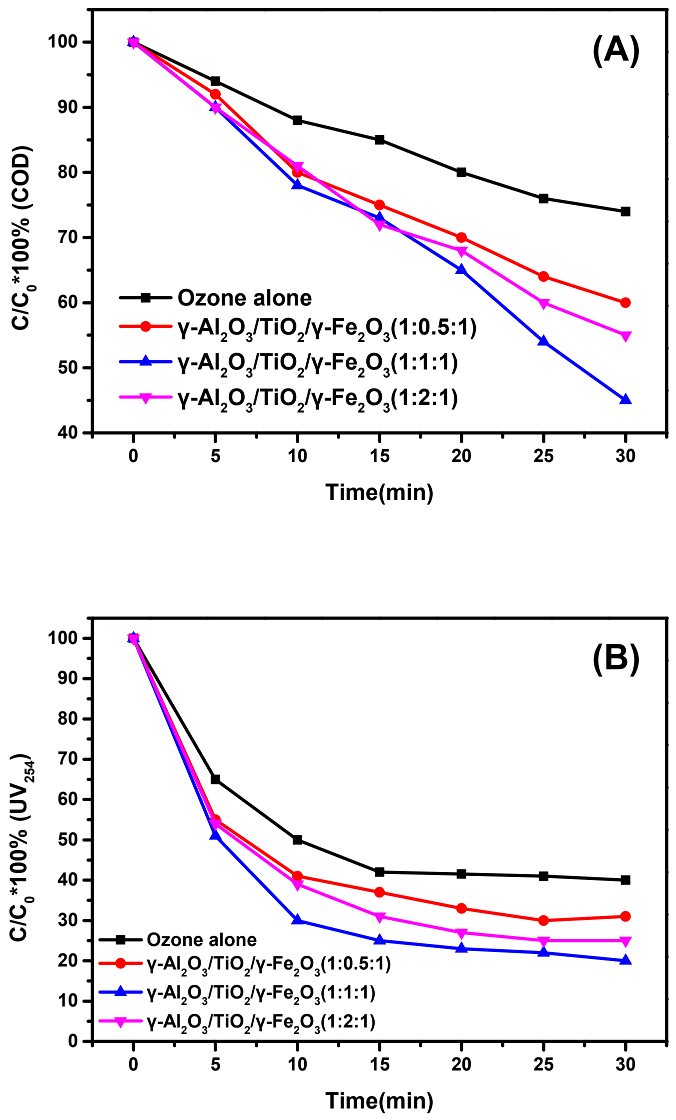

Figure S1. Removal efficiency of COD (A) and UV_{254}(B) in actual wastewater.

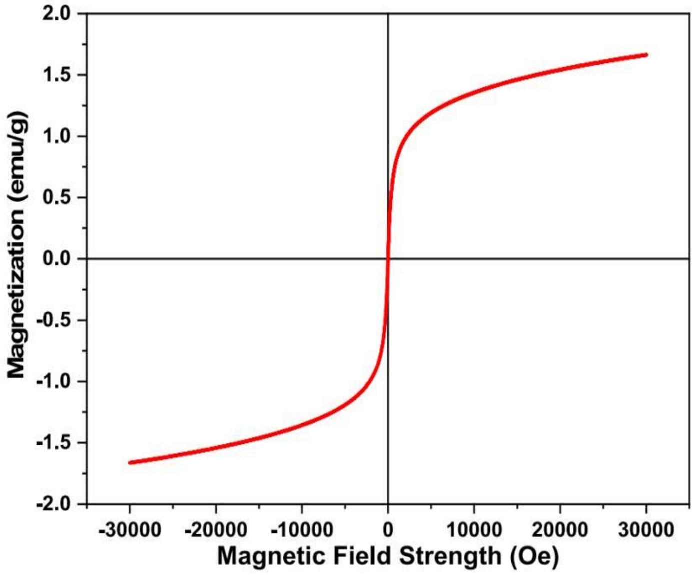

Figure S2. VSM profile of γ-Al2O3/TiO2/γ-Fe2O3.

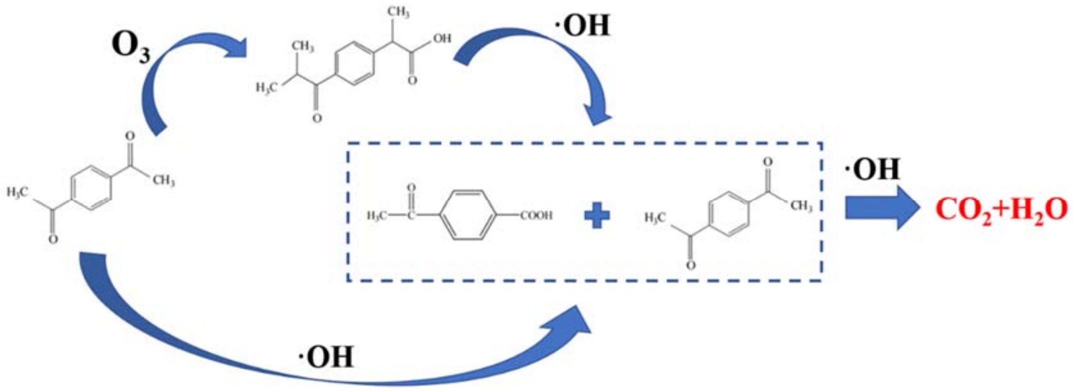

Figure S3. Path of degradation under γ-Al2O3/TiO2/γ-Fe2O3 and ozone.

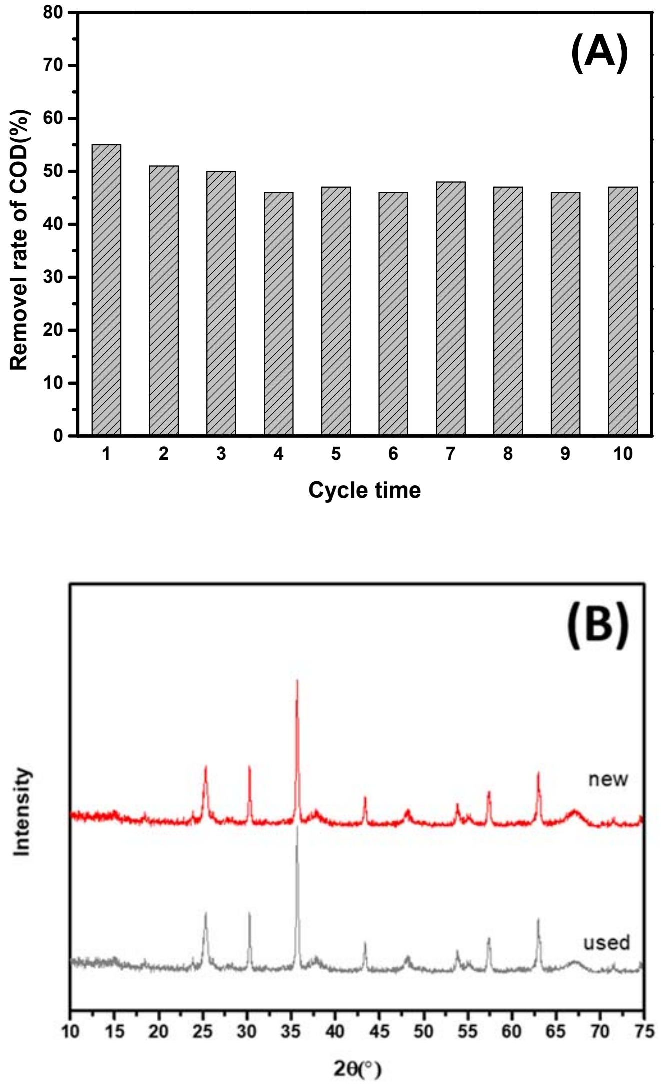

Figure S4. Catalyst cycle experiment (A) and XRD patterns before and after catalyst use (B).

Table S1. Analysis results of main organics by GC-MS.

Name of organic matter	Molecular weight	Raw water	Water sample after treatment
N-butylisoctyl phthalate	334.45	10.21	ND
Isopropyl formate	88.10	0.43	ND
Acrylamide	71.08	1.21	ND
Anthracene	178.22	3.21	ND
Pyrene	202.26	5.21	ND
Bisphenol A	228.32	0.13	ND
1-Ethynyl cyclohexene	106.17	0.54	ND
Sulfamethoxazole	253.28	0.18	ND
Fluoranthene	202.25	2.31	ND
Dimethyl phthalate	194.18	10.31	12.31
Cinnamyl aldehyde	132.16	8.12	10.12
Triethyl	276.28	ND	0.31
2,6, 11-Trimethylun-decane	208.34	ND	1.23
2-Butyloctanol	186.33	ND	2.31
3-Methyl-5-propyl non-ane	184.36	ND	1.53
